# Behavioral detection of intra-cortical microstimulation in the primary and secondary auditory cortex of cats

**DOI:** 10.3389/fnsys.2015.00061

**Published:** 2015-04-27

**Authors:** Zhenling Zhao, Yongchun Liu, Lanlan Ma, Yu Sato, Ling Qin

**Affiliations:** ^1^Department of Physiology, Interdisciplinary Graduate School of Medicine and Engineering, University of YamanashiChuo, Yamanashi, Japan; ^2^Department of Physiology, China Medical UniversityShenyang, China

**Keywords:** neural activation, auditory pathways, sensory cortex, pure tone, noise

## Abstract

Although neural responses to sound stimuli have been thoroughly investigated in various areas of the auditory cortex, the results electrophysiological recordings cannot establish a causal link between neural activation and brain function. Electrical microstimulation, which can selectively perturb neural activity in specific parts of the nervous system, is an important tool for exploring the organization and function of brain circuitry. To date, the studies describing the behavioral effects of electrical stimulation have largely been conducted in the primary auditory cortex. In this study, to investigate the potential differences in the effects of electrical stimulation on different cortical areas, we measured the behavioral performance of cats in detecting intra-cortical microstimulation (ICMS) delivered in the primary and secondary auditory fields (A1 and A2, respectively). After being trained to perform a Go/No-Go task cued by sounds, we found that cats could also learn to perform the task cued by ICMS; furthermore, the detection of the ICMS was similarly sensitive in A1 and A2. Presenting wideband noise together with ICMS substantially decreased the performance of cats in detecting ICMS in A1 and A2, consistent with a noise masking effect on the sensation elicited by the ICMS. In contrast, presenting ICMS with pure-tones in the spectral receptive field of the electrode-implanted cortical site reduced ICMS detection performance in A1 but not A2. Therefore, activation of A1 and A2 neurons may produce different qualities of sensation. Overall, our study revealed that ICMS-induced neural activity could be easily integrated into an animal’s behavioral decision process and had an implication for the development of cortical auditory prosthetics.

## Introduction

It has been well established that the auditory cerebral cortex is subdivided into a primary area (A1) and multiple non-primary areas (Read et al., 2002; Winer and Lee, 2007; Hackett et al., 2014). Previous electrophysiological research has demonstrated the response properties of neurons subjected to various sound stimuli in these areas and has yielded a greater understanding of auditory cortical circuitry. For example, neurons in the nonprimary areas are known to have more complicated response properties than those in A1 (Schreiner and Cynader, 1984; Rauschecker et al., 1995; Recanzone et al., 2000; Rauschecker and Tian, 2004), which suggests that acoustic information is hierarchically processed along the auditory pathway. However, it is difficult to establish causal relationships between neuronal activity and perception using only electrophysiological recordings. Sound stimuli cannot be used to investigate how signals in different parts of the cortex influence behavior because any given sound stimulus could activate thousands or millions of neurons distributed across many cortical areas, thereby making it impossible to attribute behavioral consequences to specific neurons.

As an alternative to sound stimulation, electrical microstimulation can be used to better understand the brain’s natural circuitry by perturbing the circuitry to generate percepts (Stanley, 2013). The ability to perturb activity within a system can provide important insights into the contribution of its components. Several previous studies have shown that animals can detect a focal stimulation of A1 with a weak electric current (intra-cortical microstimulation or ICMS; Otto et al., 2005; Deliano et al., 2009; Wang et al., 2012). Additionally, animals can be trained to discriminate stimulations presented through two spatially separated electrodes in A1 (Otto et al., 2005; Yang et al., 2008). However, the effects of ICMS have primarily been studied in A1 of animals; the effects of ICMS on the nonprimary auditory areas remain largely unknown.

To directly explore whether artificially activating different cortical areas can generate different effects, we measured the behavior of cats to detect electrical microstimulation in A1 and the second auditory field (A2). The A1 and A2 in cats are exposed at the surface of brain and are, therefore, conveniently located for the implantation of microelectrodes. In the brain, A1 is surrounded by the suprasylvian sulcus, anterior ectosylvian sulcus (AES), and posterior ectosylvian sulcus (PES), and A2 laterally borders with A1. A1 contains a systematically organized map of characteristic frequency (CF, the most sensitive frequency for a neuron), whereas A2 does not have a precise CF map, belonging to the non-tonotopic areas. In this study, as well as measuring the capability of cats to detect ICMS applied in A1 and A2, we also estimated the quality of the ICMS-evoked sensation by testing whether wideband noise and pure-tones could interfere with cats’ behavioral responses to ICMS. Our results provide new insights into the functional differentiation of primary and nonprimary regions in the auditory cortex.

## Materials and Methods

All animal work was carried out in strict accordance with the recommendations in the Guide for the Care and Use of Laboratory Animals of the National Institutes of Health. The Committee on the Ethics of Animal Experiments of the University of Yamanashi approved the protocol (permit number No.19–15). All surgery was performed under sodium pentobarbital anesthesia, and all efforts were made to minimize suffering.

### Apparatus

The behavioral experiments were conducted in a custom-built acoustically-transparent behavioral cage (54 × 44 × 49 cm) that was placed in an electrically-shielded and sound-attenuated chamber. The cats were able to move freely in the cage, and a video camera and photoelectric sensors were used to monitor their position and movement. Custom-built software implemented in the MATLAB (Mathworks) environment was used to interact with the apparatus via digital input–output hardware (PCI-6052E; National Instruments). Sound signals were digitally created by using custom-built software, generated with a D/A converter at a sampling rate of 100 kHz, and then passed through an amplifier (PMA-2000; Denon). During the behavioral experiments, acoustic stimuli were delivered via a pair of speakers (K701; AKG) placed outside the grid walls of the behavior cage. Sound calibration was conducted using a Bruel and Kjaer 1/2″ condenser microphone with a preamplifier 2669 positioned 1 cm in front of the earphone. Sound pressure level (SPL) was expressed in decibels relative to 20 µPa. The system frequency transfer function was flat up to 32 kHz (± 6 dB SPL). To examine the tonotopy of the auditory cortex in the anesthetized surgery, the speakers were placed 2 cm from the auricle of the cats.

### Pretraining of the Behavioral Paradigm

Before the implantation of electrodes, we first trained the cats to perform a Go/No-Go task to detect an acoustic stimulus. Before training, the cats were deprived of food until they reached 70–80% of their free-feeding body weight but had free access to water. The cats were first trained to lick a metal pipe when a sound was presented (tone burst: 3.2 kHz, 50 ms, and 55 dB SPL) in order to obtain a drop of liquid food, and they learned this paradigm within 1 week. Subsequently, the cats were trained to use their head to block a photoelectric sensor for at least 3 s to trigger the onset of a sound stimulus; in general, it took 2–4 weeks for the cats to become sufficiently familiar with this method and for a trial to be initiated. Next we trained the cats using target-present and target-absent trials (catch trials). Specifically, they were required to lick a metal pipe when a tone was presented (hit) and not to lick when the tone was absent (correct rejection). There were also two types of error response: licking when a target was absent (false alarm) and not licking when the target was presented (miss). In these trials, subjects were positively reinforced for the hit response only, and a punishment was not given if the subject responded in target-absent trials. By gradually decreasing and increasing the proportion of targets present and absent, respectively, the animals learned not to respond during target-absent trials and waited for a target to be presented, thereby obtaining rewards more efficiently. Training was conducted continuously over 5 days per week, and a cat could actively perform 200–300 trials per day, divided into 2–3 sessions.

To compare the subjects’ performance across sessions, we calculated an “adjusted measure” of the proportion of correct responses: p(correct) = [p(hits) + (1 − p(false alarms))]/2 × 100%. If the subject showed a Go response in all target trials [p(hits) = 100%] but not in any target-absent trials [p(false alarms) = 0], their responses were 100% correct, indicating perfect discrimination. The ratio of target-present to target-absent trials was initially set at 80:20, and gradually decreased to 50:50 once a cat’s responses were >75% correct. Cats required 2–4 months to establish a stable performance of ≥75% correct responses, which was defined by discrimination of target-present or target-absent trials at a ratio of 50:50 for five consecutive sessions. Once their performance was stable, the cat received surgery for electrode implantation.

### Surgical Preparation, Electrode Implantation, and Histology

Animal preparation and electrode implantation procedures were similar to those used in our previous experiments (Dong et al., 2011, 2013; Wang et al., 2012; Zhang et al., 2012). Briefly, cats were anesthetized with sodium pentobarbital (30 mg/kg) and fixed to a stereotaxic frame (SN-3N; Narishige). According to stereotaxic coordinates, we marked the positions of the AES and PES on the bone surface. The main part of the cats’ A1 and A2 was located between the AES and PES (Winer and Lee, 2007; Lee and Winer, 2008; Mellott et al., 2010). Four small holes were drilled over the occipital bone and fine jeweler’s screws were inserted to serve as an anchor for a metal block that was cemented to the skull with dental acrylic. After the cement had hardened, the cats’ heads were held through a metal block and the ear bars were removed. We then drilled several small holes (diameter: 0.5 mm) in the temporal bone above the potential location of the auditory cortex. A tungsten microelectrode (diameter: 250 µm; impedance: 2–5 MΩ at 1 kHz; FHC Inc.) was advanced into the cortex using a micromanipulator to examine the local field potential (LFP) in response to tonal stimuli at each site. According to the characteristics of the tonotopic gradient, we identified the locations of A1 and A2, and then implanted four microwires at these sites (two at A1, two at A2). The microwires comprised Teflon-insulated 50-µm diameter tungsten wires (part #795500; A-M Systems, Carlsborg, WA) running inside polyamide guide tubes with a 225-µm internal diameter (part #822200; A-M Systems). The tip impedance of each wire was around 0.5 MΩ at 1 kHz. The microwires were implanted through four small holes (diameter: <1 mm) separately opened on the bone. Each microwire was lowered into position using a custom-made manipulator so that the ends of the guide tube rested just above the dura mater over the cortex. The guide tube was fixed on the bone surface using dental acrylic, and then the wire was further inserted into the cortex until the tips of the electrodes were 1–2 mm below the dura. Finally, a plastic casing was attached with skull screws and cement to protect the implanted electrodes.

At the end of the experiments, the animal was deeply anesthetized and perfused with 10% formalin. The cerebral cortex was cut into coronal sections (100 µm slice) and stained with neutral red. The implantation sites were confirmed according to the lesions caused by the electrode tips.

### Identifying A1 and A2 by Recording LFPs Driven by Pure-Tones

To search for suitable sites for microwires prior to implantation, we recorded tone-evoked LFPs at several cortical sites through a tungsten electrode. The electrode signals were fed into a digital signal processing module (RX-7; TDT), and band-pass was filtered between 10 and 100 Hz to obtain spike-free signals of ongoing LFPs. We applied a set of pure-tone bursts (0.1, 0.2, 0.4, 0.8, 1.6, 3.2, 6.4, 12.8, and 25.6 kHz) to approximately evaluate the frequency tuning properties of each site. The time constraints of surgery necessitated characterizing neural responses with as few stimulus presentations as possible. The pure-tone bursts at different frequencies (160 ms duration, 5 ms linear rise/fall time, and 55 dB SPL) were randomly interleaved and repeated 10 times with inter-stimulus-intervals of >1 s. The amplitude of tone-evoked responses in the LFPs was represented by the maximum deflection during the time window, which began at the stimulus onset and ended 50 ms after the stimulus offset. Response amplitude was plotted against frequency to construct a tuning curve (Figures 4C,D). The best frequency (BF) was estimated as the frequency that corresponded to the tuning curve maximum. The bandwidth (BW) of the tuning curve was determined by measuring the peak width at half-maximum height (Figure 4C, horizontal line). The area that showed a tonotopic map (BF ordered from low to high frequency in the posterior-anterior axis) was identified as A1; the non-tonotopic area lateral to A1 was identified as A2 (Carrasco and Lomber, 2010).

### ICMS Procedures

**Figure 1:**
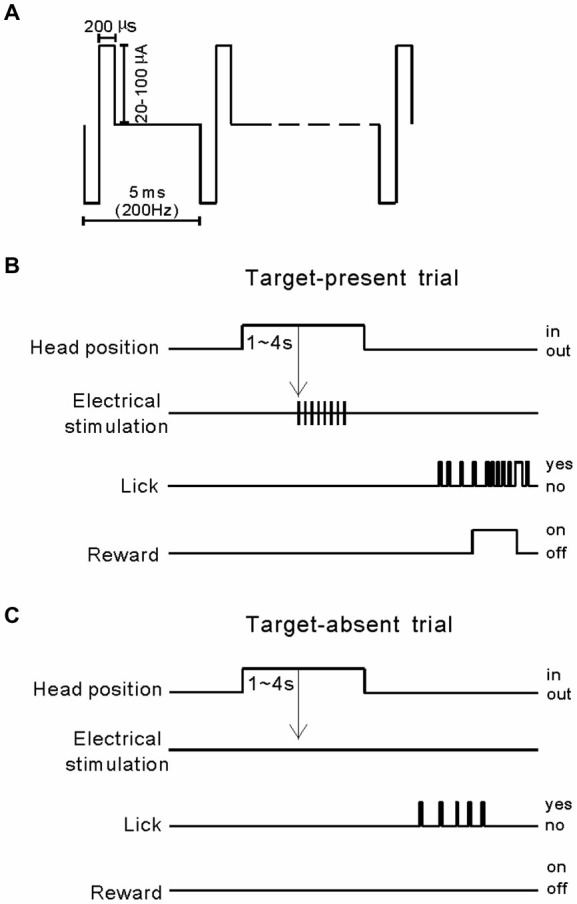
**Intra-cortical microstimulation (ICMS) and the behavioral paradigm. (A)** Stimulation pulses were delivered biphasically, with a cathodic phase preceding an anodic phase of equal amplitude. Pulse width and frequency were kept constant at 200 µs and 100 Hz, respectively. With the exception of tasks where detection sensitivities were measured, current amplitudes were always held constant at 80 µA. To measure detection sensitivities, we varied the stimulation current in the range 20–100 µA. **(B)** Target-present trial: after a cat’s head had been fixed in position for 1–4 s, a train of ICMS was presented and the cat licked a metal tube to obtain a food reward. **(C)** Target-absent trial: electrical stimulation output was turned off to measure the rate of voluntarily licking.

ICMS experiments began 2 weeks after electrode implantation. The electrical stimulus was a train of constant current, with 80 µA (pulse amplitude) and 200 µs (pulse duration) bi-phasic pulses delivered at 200 Hz for 100 ms as illustrated in Figure 1A. Previously, we proved that these were safe and effective ICMS settings, which evoked behavioral responses in cats (Wang et al., 2012). We trained the cats to make Go responses when the electrical stimulation was presented through one of the implanted electrodes (Figure 1B), and No-Go responses when the electrical stimulation was absent (Figure 1C). Because the cats were well trained in performing the behavioral task before surgery, after several sessions of training they quickly achieved >75% correct performance. After completing the training paradigm, we tested the cats using three experiments. Experiment 1 was a 100-trial session including 50 ICMS-absent trials and 10 trials each of 20, 40, 60, 80, and 100 µA pulse amplitude. This experiment was designed to test the cats’ performance in ICMS detection at different intensities in a silent environment. The aim of experiment 2 was to study the masking effect of noise on ICMS detection by presenting 50 trials of noise and ICMS (target-present) and 50 trials of noise (target-absent, Figure 3A). The duration of the noise was 320 ms with a 5 ms linear ramp, and it began 160 ms before ICMS delivery. The noise signal was generated by the MATLAB program using inverse fast Fourier transform (iFFT), which covered a frequency range from 0.1 to 32 kHz with random phases. The root mean square level of noise matched a peak-to-peak level of 10, 30, or 50 dB SPL 4 kHz pure-tone. The ICMS was presented at 80 µA and 100 Hz, and for 200 ms. The cats were rewarded for correct detection of the ICMS but not for the competing sound that was included in every trial. In experiment 3, we replaced the noise with pure-tone bursts (320 ms duration, 5 ms linear ramp, and 50 dB SPL) to test whether ICMS-evoked sensation could be disturbed by pure-tones (Figure 5A). The frequency of pure-tone was set at the BF and the BF ± 1 octave of the electrode-implanted site.

## Results

This study was conducted on six adult male cats. First, we implanted four electrodes in one hemisphere of the A1 and A2 (two electrodes in each area, with a distance >1 mm between the electrodes) and conducted the psychophysical experiments. Subsequently, the cat received further surgery to withdraw the original electrodes and implant new electrodes on the other hemisphere. In total, 48 electrodes (4 electrodes × 2 hemispheres × 6 animals) were implanted. The order of implantation was counterbalanced across animals: left then right hemisphere for half the animals, right then left for the other half. Because there was no significant difference between the data for the left and right hemispheres, we pooled these data and analyzed them together. The order of the stimulation site was also counterbalanced across animals: A1 then A2 for half the animals, A2 then A1 for the other half. For the psychophysical experiments, we first trained the cats to perform a Go/No-Go task to detect 80 µA ICMS delivered through each electrode (Figure 1). Before the implantation surgery, the cats had already learned the rules of Go/No-Go task cued by sound stimuli; thus, theoretically, if ICMS can evoke a stable sensation, they should quickly learn to perform the task cued by ICMS. Therefore, we let cats practice for 10 sessions (i.e., 1, 000 trials) to detect the ICMS at each electrode. If they achieved a high detection performance (>75% correct) within the 1, 000 practice trials, the electrode was deemed effective and used for further experiments. Following practice trials, 39 of the 48 electrodes (21 in A1 and 18 in A2) were found to be effective. There was no significant difference between the mean number of trials required to reach >75% correct detection in the groups of electrodes in A1 and A2 (595 ± 238 vs. 683 ± 204; *p* = 0.23, Student’s *t*-test). The remaining electrodes (*n* = 9) were deemed ineffective and excluded from further experiments because they might not have been successfully implanted or could not evoke a sensation without extensive training. For each of the successfully implanted electrodes, we conducted the three psychophysical experiments described in Section *Materials and methods* once.

### Experiment 1: Detection of ICMS in A1 and A2

**Figure 2:**
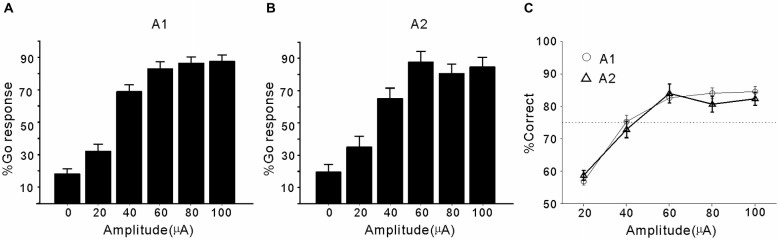
**Performance of cats in detecting ICMS in A1 and A2. (A)** Mean percentage of Go responses plotted against the pulse amplitude of ICMS in A1. Error bars represent SE across the electrodes. The % Go response to trials with 0 µA amplitude stimulation indicates the percentage of false alarms. **(B)** Mean percentage of Go responses plotted against the pulse amplitude of ICMS in A2. **(C)** Mean percentage of correct detection across the electrodes in A1 or A2. Error bars represent SE. Horizontal line indicates the level of 75% correct detection.

The Go responses to ICMS in A1 and A2 (mean percentage) are presented in Figures 2A,B, respectively. When the pulse amplitude of the ICMS was zero (corresponding to target-absent trials), the Go response (false alarm rate) was <20%. As the impulse amplitude increased from 20 to 100 µA, the Go response (hit rate) increased from 30% to >80%. For a comparison of data from different brain areas, we constructed a psychometric function by plotting the percentage of correct detection (calculated from the false alarm rate and hit rate, see Section *Materials and methods*) against pulse amplitude (Figure 2C). In both A1 and A2, the percentage of correct responses showed a monotonic increase with increasing pulse amplitude, and 75% correct detection was achieved at a 40-µA pulse amplitude. There was no significant difference in the percentage of correct responses between A1 and A2 at any of the tested pulse amplitudes (*p* > 0.05, Student’s *t*-test). Therefore, cats showed similar sensitivity to ICMS applied in A1 and A2.

### Experiment 2: Effects of Noise Presentation on the Detection of ICMS in A1 and A2

**Figure 3:**
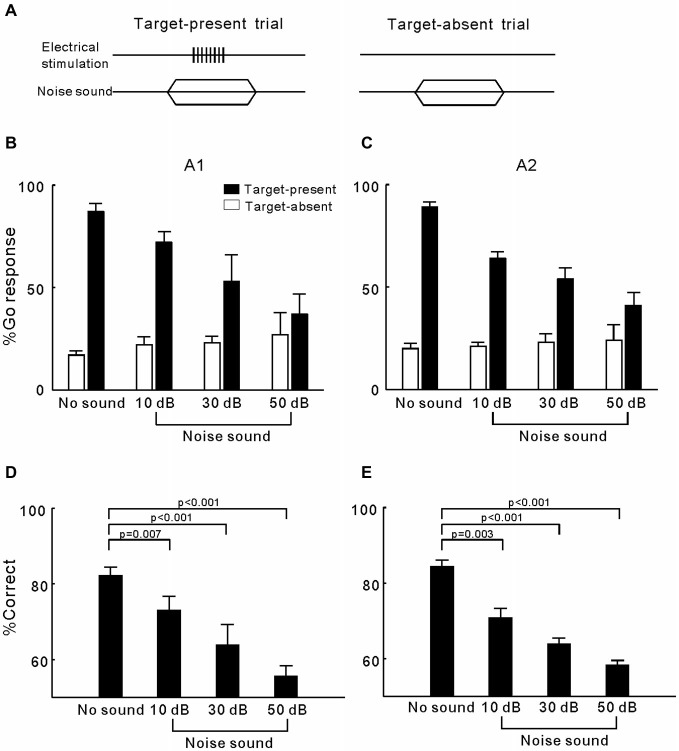
**Detection of ICMS presented together with wideband noise. (A)** Presenting both ICMS and noise in a target-present trial but only noise in a target-absent trial. **(B)** Percentage of Go responses in each Go/No-Go task for detection of ICMS in A1. Open and filled bars represent the mean percentage of Go responses in the target-absent and target-present trials, respectively. Error bars represent SE. **(C)** Percentage of Go responses in each Go/No-Go task for detection of ICMS in A2. **(D)** Mean and SE for the percentage of correct ICMS detection in A1 when presented together with different levels of noise. P values were obtained using ANOVA with a *post hoc* Student’s *t*-test. **(E)** Mean and SE for the percentage of correct ICMS detection in A2.

In experiment 2, we tested whether simultaneously presenting a wideband noise sound interfered with the cats’ ICMS detection behavior (Figure 3A). For each electrode, we tested the masking effects of a 10, 30, and 50 dB SPL noise on the detection of 80 µA ICMS. The mean percentage of Go responses across the 21 A1 electrodes and 18 A2 electrodes are displayed in Figures 3B,C, respectively. In both areas, presenting a masking noise substantially reduced the percentage of Go responses in the target trials; moreover, as the masking noise became louder, the percentage of Go responses decreased. In contrast, the percentage of Go responses in target-absent trials was less affected by masking noises. The results were transformed into the percentage of correct detection (Figures 3D,E) to make statistical comparisons between different experiment conditions. ANOVA tests revealed a significant difference between the mean percentage of correct responses when using different conditions in both A1 and A2 (*p* < 0.0001). *Post hoc* comparisons showed that correct detection in all of the noise masking trials was significantly lower than in the trials without masking conditions (*p* < 0.001). These results indicate that the cats had difficulty in detecting the ICMS when a masking noise was also presented.

### Experiment 3: Effects of Pure-Tone Presentation on the Detection of ICMS in A1 and A2

**Figure 4:**
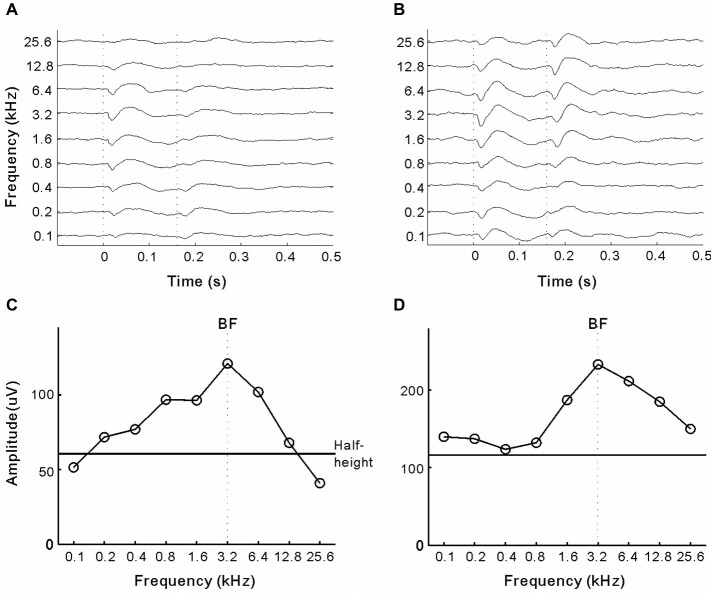
**LFPs of two example sites. (A,B)** LFPs driven by pure-tone stimuli in the A1 and A2 sites, respectively. Each line represents a local field potential (LFP) averaged across 10 repetitions of one tonal frequency. Vertical lines mark the onset and offset of sound stimuli. **(C,D)** The amplitude of LFP (peak-to-peak value) against tonal frequency. Vertical line marks the best frequency (BF), at which LFP reaches the maximum amplitude. Horizontal line marks the height of half-maximum amplitude.

**Figure 5:**
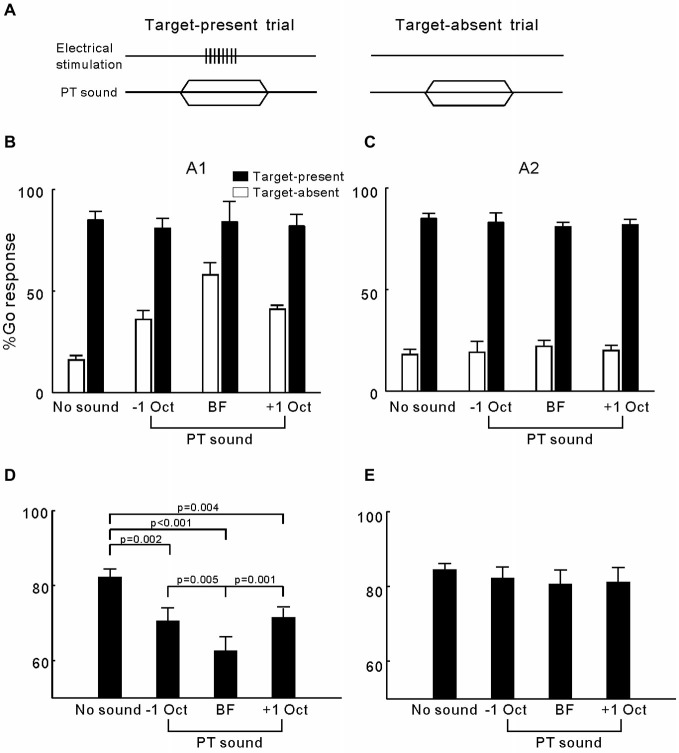
**Detection of ICMS presented together with pure-tones. (A)** Presenting both ICMS and pure-tone in a target-present trial but only pure-tone in a target-absent trial. **(B)** Percentage of Go responses in each Go/No-Go task for detection of ICMS in A1. Open and filled bars represent the mean percentage of Go responses in the target- absent and target-present trials, respectively. Error bars represent SE. **(C)** Percentage Go of responses in each Go/No-Go task for detection of ICMS in A2. **(D)** Mean and SE for the percentage of correct ICMS detection in A1 when presented together with different frequencies of pure-tones. *P* values were obtained using ANOVA with a *post hoc* Student’s *t*-test. **(E)** Mean and SE for the percentage of correct ICMS detection in A2.

In experiment 3, we investigated whether presenting pure-tones could interrupt the cats’ ICMS detection behavior. For each electrode, we estimated the BF of the cortical site on the basis of tone-driven LFPs. Figures 4A,B show the LFPs of the two representative sites in A1 and A2, respectively. Tuning curves of LFP amplitude (peak-to-peak) are presented in Figures 4C,D, in which the maximum corresponds to the BF (vertical line) and BW at half-maximum height (horizontal line) was six and eight octaves. In general, the mean BW of the tuning curves for A2 sites was significantly broader than that for A1 (6.75 ± 0.29 vs. 5.70 ± 0.30 octaves, *p* = 0.014, Student’s *t*-test). For each electrode, we tested the cats’ performance in detecting ICMS when presented together with 50 dB SPL pure-tones at the BF and BF ± 1 octave (Figure 5A). The mean percentages of Go responses to ICMS in A1 groups are displayed in Figure 5B. The hit rate (the percentage of Go responses in the ICMS-present trials) was not changed by the presentation of pure-tones but the false alarm rate (percentage of Go responses in ICMS-absent trials) clearly increased. Consequently, the mean percentage of correct detection was significantly lower when pure-tone masking was present compared with conditions where masking was absent (*p* < 0.0001, ANOVA with *post hoc* Student’s *t*-test, Figure 5D). Additionally, when pure-tones were presented at the BF of the electrode-implanted site, they produced a stronger disturbance effect than pure-tones that were further away from the BF. This result implies that the cats’ detection of ICMS in A1 is similar to that of pure-tones at the BF of ICMS-activated neurons. In contrast to A1, the ICMS detection performance in A2 was not significantly changed by the presentation of pure-tones (Figures 5C,E), suggesting that detection of ICMS in the cats’ A2 is independent of the frequency tuning of ICMS-activated neurons.

## Discussion

The central aim of this study was to investigate the different behavioral consequences of using ICMS to activate A1 and A2. We found that cats detected ICMS with similar sensitivity in A1 and A2. Masking noise interfered with the behavioral detection of ICMS in both A1 and A2; however, presenting pure-tones at frequencies around the BF of the electrode-implanted site reduced ICMS detection performance in A1 but not A2. Therefore, ICMS in A1 and A2 may evoke different qualities of sensation.

The ability of human patients to reliably detect electrical stimulation in some parts of the cerebral cortex was documented by Penfield and Perot (1963), who showed that stimulation of the primary sensory areas produces patent modality-specific percepts. This finding is supported by many animal studies, which report behavioral responses to ICMS in the primary somatosensory (Butovas and Schwarz, 2007), auditory (Rousche and Normann, 1999; Wang et al., 2012), and visual cortexes (Bartlett et al., 2005; DeYoe et al., 2005). However, Penfield and Perot’s results showed that human patients rarely detected stimulation outside the primary cortical areas. In this study, we found that the cats quickly learned to detect ICMS delivered from the majority of electrodes (39/48) implanted in both A1 and A2. In order to achieve satisfactory performance (>75% correct detection), auditory training was required in daily sessions conducted over 2–4 months. However, all six cats in the study were able to immediately detect the microstimulation cues, achieving an accurate performance within 1, 000 practice trials. This suggests that cortical microstimulation is a robust method that can be easily integrated into an animal’s behavioral decision process. Furthermore, there was no significant difference between the practice trials for stimulus transfer in A1 and A2, indicating that neuronal signals in these areas are similarly accessible for guiding behavior. This result is consistent with the idea that ICMS in the sensory cortex can generate sensations, regardless of the hierarchical level of the stimulated area (Murphey and Maunsell, 2007).

Previous studies suggest that animals gradually improve their detection of ICMS as their level of practice increases. For example, in initial trials a monkey could not detect a <50 µA stimulation in the V1 but after thousands of practice trials, detection thresholds approached 6 µA (Ni and Maunsell, 2010). In this study, we focused on the potential differences between the effects of ICMS in A1 and A2. To exclude the effects of training, we measured the cats’ ICMS detection performance using different test conditions that were applied soon after they had learned to perform the ICMS-cued task. We found that there was no significant difference between the cats’ performance when detecting various amplitudes of ICMS in A1 and A2, and all performance curves crossed the 75% correct detection level at around a 40-µA pulse amplitude. Therefore, the sensations evoked by ICMS in A1 and A2 had similar saliency. This result is consistent with the findings of Murphey and Maunsell (2007), which indicated that sensitivities for detecting electrical microstimulation are similar across different areas of the visual cortex.

Some previous studies have suggested that electrical stimulation on the sensory cortical areas can produce modality-specific percepts. For example, stimulation of the surface of the V1 produces the sensation of a small point of light or phosphine (Penfield and Perot, 1963; Brindley and Lewin, 1968; Lee et al., 2000). Microstimulating the V1 in monkeys while presenting a small visual stimulus affects both the probability that the animal will make a saccade to that stimulus and the latency of the response (Tehovnik et al., 2002). Additionally, microstimulation of the somatosensory cortex in monkeys has a similar effect to a mechanical vibration applied to the finger in tactile discrimination (Romo et al., 1998). In this study, we found that noise presented concurrently with ICMS reduced the detection of ICMS in both A1 and A2, which was indicated by a decreased hit rate with no change in the false alarm rate. This result is consistent with an auditory masking effect. Furthermore, we found that pure-tones presented with ICMS reduced ICMS detection in A1. In contrast to the results of the noise masking experiment, pure-tone presentation increased the false alarm rate but did not change the hit rate. Pure-tones corresponding to the BF of the stimulation site also evoked a higher false alarm rate than those that were higher or lower than the BF. Because the cats were only rewarded for correct detection of the ICMS and not the competing sound, the increased false alarm rate indicates that the cats perceived pure-tones as ICMS. Therefore, ICMS-evoked activity of A1 neurons may be processed by higher-order stages as a tone-evoked activity. This result suggests that ICMS in A1 can have an auditory-specific effect. It is noteworthy that our present result was obtained using Go/No-Go detection tasks, rather than a discrimination task that directly evaluated the difference between the sensations elicited by ICMS and sound. We cannot rule out the possibility that the cats learned to receive a reward by detecting some non-selective effect of the ICMS. Therefore, it is premature to draw a firm conclusion that ICMS can evoke an auditory specific percept.

We found that detection of ICMS in A2 was not affected by pure-tone presentation. The observed difference in behavioral responses between A1 and A2 may be attributable to the difference in the physiological characteristics of A1 and A2 neurons. The neural responses of A1 to various simple and complex sounds have been well examined in both anesthetized (Schreiner and Urbas, 1988; Eggermont, 1991; Schreiner et al., 1992; Sutter and Schreiner, 1995; Noreña and Eggermont, 2002) and awake cats (Qin et al., 2008a,b; Ma et al., 2013). A1 neurons usually respond to a specific range of tone frequencies (spectral receptive field)., and the tuning frequency of A1 neurons is ordered from low to high in the posterior-anterior axis, constructing a clear tonotopic map. According to published data (Tehovnik and Slocum, 2007), the 80 µA amplitude of electrical stimulation that we used in this study may activate a local population of neurons lying within a few hundred microns of the electrode tip. Because the adjacent neurons in A1 have a similar spectral receptive field, activating these neurons may generate a relatively homogenous sensation. In a similar manner, studies on the V1 also suggest that electrical stimulation produces a visual sensation corresponding to the visual receptive field of neurons at the site of stimulation (Penfield and Perot, 1963; Brindley and Lewin, 1968; Lee et al., 2000; Tehovnik et al., 2004, 2005). Conversely, electrophysiological studies have shown that A2 neurons have a broader spectral receptive field and no clear frequency topography (Schreiner and Cynader, 1984; Schreiner and Urbas, 1988; Eggermont, 1998). Therefore, ICMS in A2 may activate a population of neurons with different receptive fields, which then produce a heterogeneous auditory sensation. Our result provides evidence to support the idea that A2 is specialized in processing types of acoustic information that are more complex (Schreiner and Cynader, 1984).

## Conflict of Interest Statement

The authors declare that the research was conducted in the absence of any commercial or financial relationships that could be construed as a potential conflict of interest.
